# How Crawling and Manual Object Exploration are Related to the Mental Rotation Abilities of 9-Month-Old Infants

**DOI:** 10.3389/fpsyg.2013.00097

**Published:** 2013-03-04

**Authors:** Gudrun Schwarzer, Claudia Freitag, Nina Schum

**Affiliations:** ^1^University of GiessenGiessen, Germany

**Keywords:** mental rotation, self-produced locomotion, manual exploration, infancy, crawling

## Abstract

The present experiment examined whether the mental rotation ability of 9-month-old infants was related to their abilities to crawl and manually explore objects. Forty-eight 9-month-old infants were tested; half of them had been crawling for an average of 9.3 weeks. The infants were habituated to a video of a simplified Shepard–Metzler object rotating back and forth through a 240° angle around the longitudinal axis of the object. They were tested with videos of the same object rotating through a previously unseen 120° angle and with a mirror image of the display. All of the infants also participated in a manual object exploration task, in which they freely explored five toy blocks. The results showed that the crawlers looked significantly longer at the novel (mirror) object than at the familiar object, independent of their manual exploration scores. The non-crawlers looking times, in contrast, were influenced by the manual exploration scores. The infants who did not spontaneously explore the toy blocks tended to show a familiarity preference, whereas those who explored the toy blocks preferred to look at the novel object. Thus, all of the infants were able to master the mental rotation task but it seemed to be the most complex process for infants who had no crawling experience and who did not spontaneously explore objects.

## Introduction

Mental rotation refers to the ability to rotate mental representations of two-dimensional and three-dimensional objects (Linn and Petersen, 1985). This ability is crucial even for infants because from soon after birth they are confronted with moving and rotating objects in their natural environment. The question arises regarding the developmental factors that are relevant to infant mental rotation ability. Recent studies of young infants showed that gender plays a crucial role, indicating an advantage for male infants (Moore and Johnson, 2008, 2011; Quinn and Liben, 2008). Studies with older infants suggested that motor experience might be relevant for mental rotation ability. Campos et al. (2000) provided evidence that self-produced locomotion, such as crawling, is relevant for infant visual-cognitive ability in general, and Schwarzer et al. (2012) demonstrated a specific association between infant crawling and mental rotation ability. In addition, recent research has suggested that sophisticated manual object explorations, including rotations, fingerings, and transfers of objects (Soska et al., 2010), and prior manual object experience might be associated with infant mental rotation ability (Möhring and Frick, in press). However, these studies did not address the question of whether a combination of both crawling and manual object exploration is related to infant mental rotation ability. Crawling around a piece of furniture, for example, or manually rotating a toy or transferring it from one hand to the other permits the infant to detect the invariant properties of objects and to recognize them from novel perspectives. Such a joint significance can be hypothesized, as both motor skills provide infants with the opportunity to view objects from different perspectives and to learn to understand different rotations of a target object as belonging to the same object. In our experiment, we aimed to investigate if and how crawling and manual object exploration skills are related to the mental rotation abilities of infants.

### Mental rotation ability in infants

To date, few studies have investigated mental rotation ability in infants. In the first studies carried out by Rochat and Hespos (Rochat and Hespos, 1996; Hespos and Rochat, 1997) infants as young as 4 months of age were required to extrapolate the trajectory of an object rotating through a 120° arc and continued its trajectory for 60 more degrees behind an occluder. When revealed at the end of the event, the object was in a probable or improbable orientation. Results showed that the infants looked longer at the improbable than at the probable orientation but only when the invisible rotation angles were relatively small. These results were interpreted as first evidence of infants’ mental rotation ability.

More recently, Quinn and Liben (2008) and Moore and Johnson (2008, 2011) conducted studies with 3- to 4-month-old and 3- and 5-month-old infants, respectively, using tasks that were closely related to the classic mental rotation task by Shepard and Metzler (1971) used with adults. In Shepard and Metzler’s (1971) task subjects viewed a two-dimensional image of a three-dimensional object and then were asked to distinguish between a novel view of the same object and its mirror image. Quinn and Liben familiarized 3- to 4-month-old infants with a series of two-dimensional images of the numeral “1” and then preference-tested them with a novel orientation of the numeral “1” paired with its mirror image. They found that boys displayed a novelty preference for the mirror image, but that girls did not. Moore and Johnson obtained similar results with 3- and 5-month-old infants. They used a simplified, three-dimensional Shepard–Metzler object and habituated the infants to this object as it rotated through a 240° angle. In test trials, infants saw the familiar object or its mirror image rotating through a previously unseen 120° angle. Only the boys differentiated between the familiar and the mirror object. The 3-month-old male infants looked significantly longer at the familiar object, while the 5-month-old male infants preferred to look at the mirror object. Thus, mental rotation seemed possible even for the 3-month-old boys, providing evidence of a gender difference in the ability to mentally rotate an object in three-dimensional space.

### Influence of motor experience on mental rotation ability

A link between motor experience and infants’ understanding of objects was suggested long ago by Piaget (1952). He argued that infants’ object understanding is based on acquired information about objects through sensorimotor experiences or actions. Piaget linked action and object representation in the sense of action being the origin of cognition. Other researchers (e.g., Gibson, 1988; Adolph et al., 1993; Bushnell and Boudreau, 1993) proposed that infants’ action systems tune their perceptual systems which allows them to gain new information about objects. Increases in infants’ activity with objects fine-tune their perceptual systems to the association between the characteristics of objects and the actions afforded by the objects.

With regard to mental rotation ability, previous research has demonstrated a link between motor experience and mental rotation ability in children. For example, in 10- to 11-year-old children, Wiedenbauer and Jansen-Osmann (2008) found that manual rotation training could improve mental rotation performance. In another study, Jansen and Heil (2010) showed that mental rotation ability was related to motor development in 5- to 6-year-old children. Children performed a standardized motor test, a paper-pencil mental rotation test, and a non-verbal reasoning test. A multiple regression analysis revealed that non-verbal reasoning and motor ability were significant and independent predictors of the children’s mental rotation performance.

Few studies focused on the relationship between motor experience and mental rotation ability, or a related ability, in infants. Recently, Schwarzer et al. (2012) investigated the relationship between the mental rotation ability of 9-month-old infants and their ability to crawl, which naturally provides infants with different views of objects. Schwarzer et al. (2012) used a similar rotation task as Moore and Johnson (2008, 2011) and revealed that crawling, but not non-crawling, infants mastered the mental rotation task. Campos et al. (1980) studied the relationship between infant crawling experience and an ability that might be related to mental rotation, the ability to recognize different shapes of objects independently of orientation, size, and color. The study found that the performance of infants who were crawling was significantly better than that of non-crawling infants.

Soska et al. (2010) have demonstrated that infant motor skills, such as sitting and manual object exploration, are related to the ability to complete non-visible parts of three-dimensional objects, which can be seen as an ability that is relevant to mental rotation. In particular, they found that visually coordinated manual object explorations, such as rotations, fingerings, and transfers, contributed to the three-dimensional object completion skill. Möhring and Frick’s (in press) results were similar. They showed that 6-month-old infants succeeded in a mental rotation task if they had the opportunity to manually explore the test object prior to the experiment. Needham (2000) also found that infants’ exploration activity with objects enhanced their ability to process objects as to detect edge assignments and to segregate spatially contiguous objects. Similarly, Perone et al. (2008) revealed that infants’ skilled activity with objects during naturalistic play was related to a more advanced understanding of objects like responding to a change in object appearance.

Such results suggest that gross and fine motor experiences, including crawling and manual exploration of objects, are related to an advanced understanding of objects and the mental rotation ability of infants in particular, but it is unclear whether and how a combination of both skills is related to mental rotation. We hypothesize that the effects of both crawling and manual object exploration are related to mental rotation ability and might interact with each other, reinforce or compensate each other, as both types of motor skills provide infants with the opportunity to view objects from different perspectives and to enhance the detection of object invariance.

**Figure 1:**
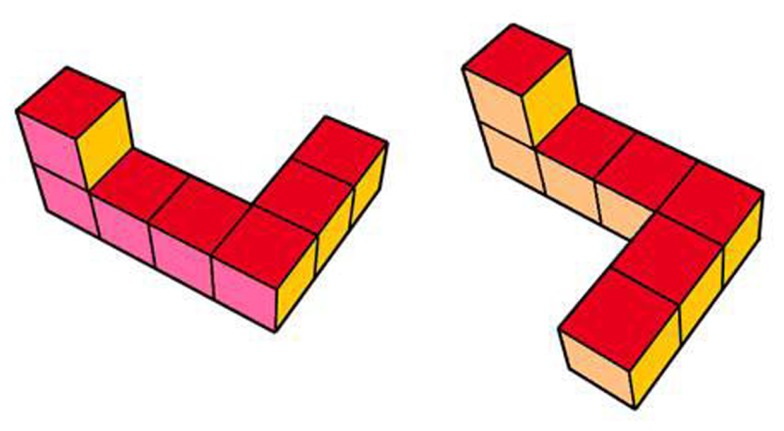
**Images of the simplified Shepard–Metzler objects, the L-object and its mirror image, the R-object, pictured on the left and right, respectively**.

In the present study we examined the extent to which mental rotation ability of 9-month-old infants was related to their crawling ability and to their ability to explore objects in a sophisticated manner. We investigated 9-month-old infants because usually approximately 30–40% of that age group is able to crawl. According to Moore and Johnson (2008, 2011) we presented 9-month-old infants with a video representation of a 3-D habituation object (Figure 1) revolving around the longitudinal axis in 3-D space, through a 240° angle. We hypothesized that infants would recognize that object in subsequent test trials in which the object revolved through a previously unseen 120° angle. After habituation, each infant saw alternating test trials presenting the original habituation object or its mirror image; in both cases, the test objects were shown revolving through the previously unseen 120° angle. We reasoned that evidence for mental rotation would be revealed in the test trials by a looking preference for the mirror-image, i.e., novel object; such a preference would imply the recognition of the other test object as the original habituation object, now only rotating through a novel angle. A preference for the mirror-image stimulus in our study could not be due to low-level stimulus characteristics (e.g., brightness, colors, etc.) because the test stimuli were identical in all those respects. We proposed that recognizing the habituation object from the new perspective would require infants to rotate a mental representation of either the habituation object or the visible test stimulus although we could not rule out that other processes such as structural description could enable infants to match the habituation object from the new perspective and the habituation object familiar from the habituation phase. In particular, we predicted that infants who were able to crawl and to manually explore objects in a sophisticated manner look longer at the mirror object than at the familiar object as both motor activities provide infants with the opportunity to view objects from different perspectives. Infants who did not show such motor abilities were expected to show no preferences for neither the familiar nor the mirror objects. We had no clear predictions whether and how the proposed effects of crawling and object exploration on infants’ mental rotation abilities interact which each other. The effects can be additive but they can also be compensatory and replace each other.

## Materials and Methods

### Participants

Participants included 58 full-term, healthy 9-month-old infants. Ten infants were excluded because of experimenter error (*N* = 3), failure to complete the task (*N* = 4), and recording errors of the manual exploration task (*N* = 3). The final sample consisted of 22 female and 26 male infants (mean age: 9 months, 5 days; range: 9–9 months, 10 days). To acquire information regarding the infants’ crawling ability we sent a movement calendar (constructed by the authors) to the parents 4 weeks before testing the infant in the lab. In the calendar, the parents were asked to report when their baby began to be capable of crawling, defined as moving in a prone position on the hand and knees for a distance of at least 2 m. We also asked the parents, when their baby was able to turn from the back to the belly, to sit, and when the baby started to crawl on the belly and for how many meters. In the case of uncertainties, we discussed the entries with the parents. Bodnarchuk and Eaton (2004) had shown that parents provide reliable reports of their infants’ attainment of motor milestones, including crawling. Twenty-four infants (12 girls and 12 boys) had been crawling for an average of 9.3 weeks. One infant crawled for 3 weeks and all other infants crawled for 4 weeks at least. According to Ueno et al. (2011) infants having at least 4 weeks of crawling experience can be classified as experienced crawlers. Twenty-four infants were not able to crawl (10 girls, 14 boys). All of the infants were from middle class families.

### Stimuli

The infants participated in a mental rotation task and an object exploration task. In the mental rotation task, stimuli presented in Schwarzer et al. (2012) were used that only slightly differed in the colors of the faces. The stimuli were 3-D models of simplified Shepard–Metzler objects, an L-object and an R-object. These two objects were mirror images of one another and are shown in Figure 1.

The faces of the L-object were medium-red when viewed from above, dark-red when viewed from below, pink when viewed from the front, ocher when viewed from the back, yellow when viewed from the right, and gold when viewed from the left (see Figure 1). The faces of the R-object were constructed accordingly. The maximum vertical and horizontal dimensions of the objects during presentations of the images were reached at 16° and 12° of the visual angle, respectively.

**Figure 2:**
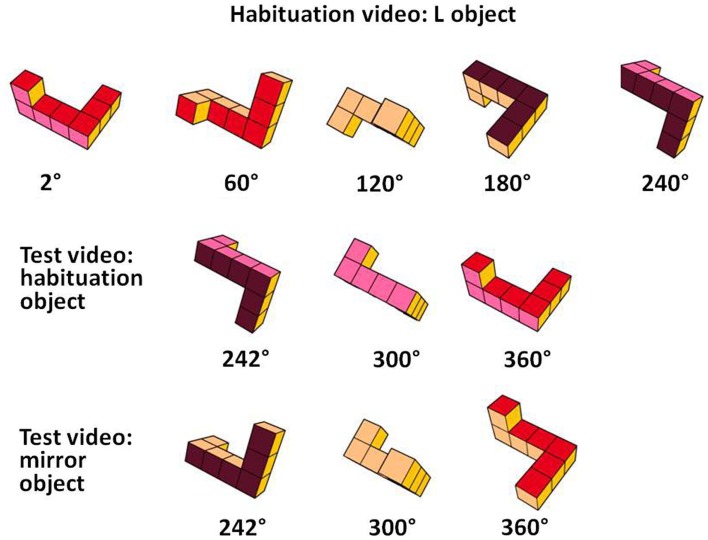
**Examples of the images presented in the habituation and test videos**. The images of the habituation video rotated back and forth through a 240° angle (2° to 240°). The images of the test videos rotated back and forth through a previously unseen 120° angle (242° to 360°).

We constructed habituation and test videos of the objects. Each video was composed of a series of images of the same object (L- or R-object) that were rotated an additional 2° around the longitudinal axis of the object, which differed 60° from the vertical axis (see Figure 2).

This series of images appeared as an object rotating at 52° per second. The habituation video comprised a rotation between 2° and 240°, and, upon reaching the maximum extent of rotation, the object appeared to reverse course, rotating back to its starting point.

The videos of the L- and R-objects used for the test videos continued the rotation of the L- and R-objects, respectively, through the previously unseen 120° arc. Together, a habituation video and its corresponding test video represented a complete 360° turn around the longitudinal axis. The objects in the test videos continuously rotated back and forth between the starting points and the maximum extent of their rotations. No orientation of either habituation stimulus was identical to any orientation of either test stimulus.

**Figure 3:**
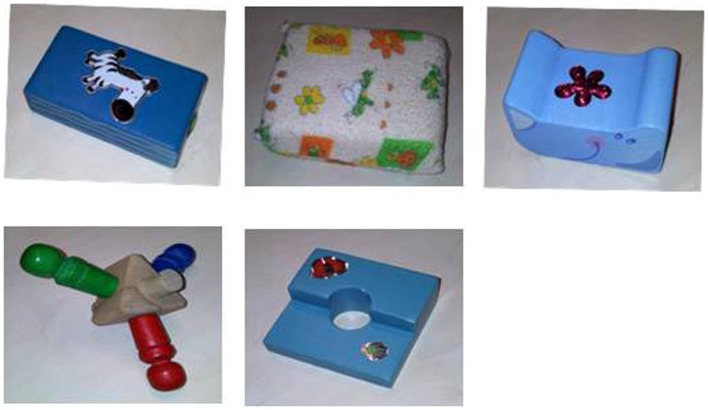
**Stimuli presented in the manual object exploration task**.

The stimuli of the manual exploration task consisted of five toy blocks (see Figure 3). They were between 6 and 10 cm wide, fit easily into the infants’ hands and were readily graspable. They were made of plastic or wood and had colorful patterns on the front and back.

### Apparatus

The mental rotation task was conducted in a rectangular cubicle with an open side that was designed to accommodate a caregiver sitting on a chair with a child on her lap. Each infant was seated on the caregiver’s lap at a distance of 60 cm from the computer monitor screen that displayed the stimuli that was inserted into the rear wall of the cubicle. To prevent parents from influencing their babies’ looking times they were asked to keep their eyes closed and to refrain from talking for the duration of the experiment. The entire session was recorded on a VCR using a low-light video camera attached to a peephole in the back of the cubicle.

The manual exploration task took place at a table where the infants were seated in a highchair. The entire session was recorded on a VCR by a camera placed in front of the infants.

### Procedure

Each infant was tested individually. The order of the mental rotation task and the manual object exploration task was counterbalanced across infants.

The mental rotation task consisted of a habituation phase and a test phase. The infants were randomly assigned to the L- or R-habituation videos. In the habituation phase, the infants were presented with a habituation video portraying the L- or R-object. Trials were accompanied by an auditory attention-getter and began when the infants looked at the monitor. Looking time to the object was recorded online by the experimenter, by pressing a button. The experimenter was naïve to the hypotheses under investigation and to the locomotion category of the infants. Each trial ended either 2 s after the experimenter released the button to indicate that the infant was no longer fixating the display or after 60 s had passed. The trial continued if the infant returned their attention to the video during the 2 s interval. The habituation phase ended when the average time fixating to the habituation video declined to 50% within three consecutive trials, compared to the average time of fixation within the first three habituation trials or when a maximum of 12 habituation trials were presented. After habituation, a series of 3 × 2 test videos presenting the habituation object and the corresponding mirror object through the previously unseen 120° arc was presented alternately. The order of presentation of the first test video (presenting the habituation object or the mirror object, see Figure 2) was counterbalanced across trials.

Trained observers who were naïve to the hypotheses under investigation recorded the time infants spent looking at the stimuli using videotapes of the sessions. The inter-observer reliability exceeded 0.9.

In the manual object exploration task, an experimenter offered the infants five objects (see Figure 3) one at a time, for one trial each, in a counterbalanced order across the sample. Each of the trials began with the experimenter presenting the object at midline. The trials lasted from the moment when the infants grasped the object until they had accumulated 40 s of spontaneous manual exploration. If infants dropped the object and did not recover it within 5 s, the experimenter offered the object again. After 40 s of accumulated play, the experimenter removed the object from infant’s hand and offered the next object. All the trials were recorded with a VCR.

A coder scored the object exploration data using the video tool virtualdub to determine the frequencies of the infants’ actions. According to the results of Soska et al. (2010), we focused our analyses on rotations, transfers, and fingerings performed while the infants looked at the objects. A rotation was scored when the infants rotated an object at least 90°; a fingering was scored when the infants moved their fingers over the surface of the object; a transfer was scored when the infants transferred an object between their hands, when both hands held the object for less than 5 s. A second coder scored 50% of the data to verify the reliability of the codes. Inter-coder reliability for rotations, fingering, and transfer exceeded 0.85.

## Results

The infants’ average looking times of the test phase to the novel (mirror image) object and the familiar object were computed, and the differences between looking times to the novel and familiar objects were calculated. Preliminary analyses examining the effect of habituation with the L- versus R-object, gender, and order of test stimulus presentation, as well as the order of the exploration task, on looking time differences revealed no reliable main effects or interactions; therefore, the data were collapsed across these variables for the following analyses.

To assess the effect of manual object exploration, we computed a sophisticated exploration score. This score included the average number of rotations, fingerings, and transfers for each infant across the five objects (see Soska et al., 2010). We divided the exploration scores into three categories. Category 0 included infants with an exploration score of 0, indicating that the infants did not spontaneously show any rotations, fingerings, or transfers. Seven infants of category 0 were crawlers and 5 non-crawlers. These infants only dropped the objects, hit them on the table or threw them away. Category 0 paralleled the category of the non-crawlers, who also did not show any signs of the target skill. Category 1 included infants whose exploration score was below 25 actions, the median of the exploration score. Eight infants of category 1 were crawlers and 9 were non-crawlers. Category 2 included infants with an exploration score that exceeded 25 actions. Nine infants of category 3 were crawlers and 10 were non-crawlers. Crawlers and non-crawlers did not differ with respect to their manual exploration scores, *t*(46) = −0.54, *p* = 0.59 [crawlers *M* = 1.08 (SD = 0.83) and non-crawlers *M* = 1.21 (SD = 0.78), respectively].

**Figure 4:**
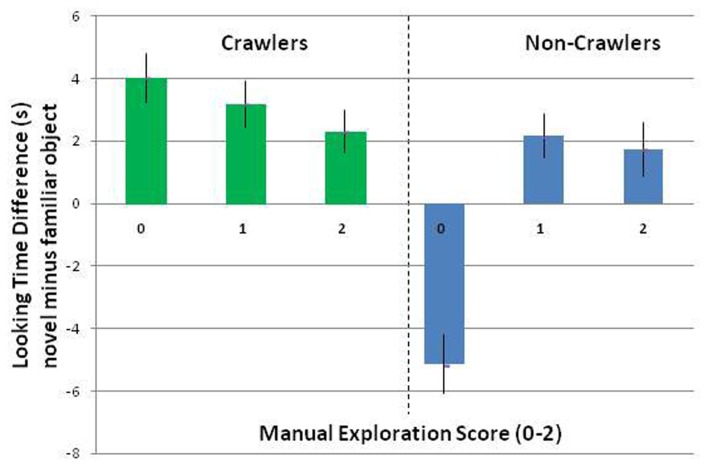
**Nine-month-olds’ mean looking time differences between novel (mirror) and familiar test objects**. Error bars indicate standard error of the mean.

An univariate ANOVA of the looking time differences between the novel and familiar objects with crawling (crawling: yes, no) and exploration score (categories: 0, 1, 2) as between subjects factors revealed two significant effects, a main effect of crawling, *F*(1, 42) = 8.23, *p* < 0.006, eta^2^ = 0.16, and a crawling × exploration score interaction, *F*(2, 42) = 4.66, *p* < 0.01, eta^2^ = 0.18, see Figure 4. The main effect of crawling revealed that the crawling infants showed longer looking time differences between the novel and familiar objects [*M* = 3.1 s (SD = 4.36)] compared to the non-crawling infants [*M* = 0.46 s (SD = 4.69)], *t*(46) = 2.02, *p* < 0.05.

The crawling × exploration score interaction indicated that the manual exploration score had no influence on the crawling infants regarding the looking time differences between the novel and familiar objects, *F*(2, 21) = 0.29, *p* = 0.75. All of the crawlers looked longer at the novel object, independent of their exploration score [difference between the novel and familiar objects for category 0: *M* = 4.03 s (SD = 3.18), for category 1: *M* = 3.18 s (SD = 4.78), for category 2: *M* = 2.31 s (SD = 5.09)]. In the non-crawlers, in contrast, the exploration score had a significant impact on the looking time differences when using a Bonferroni correction, *F*(2, 21) = 6.83, *p* < 0.005, eta^2^ = 0.39. The non-crawlers with an exploration score of 0 showed a strong preference for the familiar object [difference between novel and familiar object for category 0: *M* = −5.14 s (SD = 6.17)], whereas the group of non-crawlers with exploration scores of 1 and 2 showed a preference for looking at the novel object [Category 1: *M* = 2.17 s (SD = 3.7), Category 2: *M* = 1.7 s (SD = 2.22)].

In order to further investigate the impact of infants’ exploration score on their looking behavior, we conducted the same univariate ANOVA as previously described but divided the factor exploration scores into two categories. That procedure seemed justified as infants with exploration scores of 1 and 2 showed similar preferences for looking at the novel object. As before, category 0 included infants who did not show any transfers, rotation, or fingerings of the objects. Category 1 included all of the infants who showed sophisticated manual object exploration. The results revealed the same effects as described above, i.e., a significant effect of crawling, *F*(1, 44) = 13, *p* < 0.001, eta^2^ = 0.23, and a significant interaction between crawling and the exploration scores, *F*(1, 44) = 9.24, *p* < 0.004, eta^2^ = 0.17, but in the revised analysis, the effect of exploration score also reached significance, *F*(1, 44) = 4.38, *p* < 0.04, eta^2^ = 0.09. The infants with an exploration score of 0 showed a significantly lower looking time difference [*M* = 0.21 s (SD = 6.46)] than infants with an exploration score of 1 [*M* = 2.31 s (SD = 3.89)].

In order to analyze measures of information processing in crawling and non-crawling infants and in exploring and non-exploring infants, we compared their total looking times and number of trials within the habituation phase. We did not find any differences between the crawlers and non-crawlers regarding total looking times [crawlers: *M* = 91.36 s (SD = 52.9), non-crawlers: *M* = 92.08 s (SD = 44.03)] and number of trials [crawlers: *M* = 7 trials (SD = 2.53), non-crawlers: *M* = 6.5 trials (SD = 2.52)]. Explorers and non-explorers also did not differ in their total looking times [explorers: *M* = 92.98 s (SD = 48.59), non-explorers: *M* = 89.12 s (SD = 48.81)] and number of trials [explorers: *M* = 6.78 trials (SD = 2.61), non-explorers: *M* = 6.67 trials (SD = 2.53)].

## Discussion

The present study revealed that the mental rotation ability of 9-month-old infants was related to their crawling ability and to their spontaneous willingness to explore objects in a sophisticated manner. After habituation to different dynamically presented orientations of an object, crawling infants and infants who spontaneously explored objects were better able to generalize the habituation images of the same object in a new orientation and exhibited longer looking times to mirror images of the object. However, most importantly, the effects of crawling and manual object exploration were qualified by a significant interaction between the factors. Whereas the looking times of crawling infants to the novel (mirror) object were independent of their manual exploration skills, the looking times of non-crawling infants differed depending on their exploration scores. The non-crawling infants who did not spontaneously explore the objects preferred to look at the familiar object in a new orientation, while the non-crawling infants who spontaneously explored the objects preferred to look at the novel object.

The results are consistent with theories highlighting the fundamental role of action or motor experience in infants’ understanding of objects (e.g., Piaget, 1952; Gibson, 1988; Adolph et al., 1993; Bushnell and Boudreau, 1993; Needham, 2000). They support the notion that infants’ actions on objects alter what they attend to, perceive, remember, and process about objects. Our findings are in line with those of related studies that emphasize such interaction between motor skills and visual-cognitive abilities (Campos et al., 2000; Wiedenbauer and Jansen-Osmann, 2008; Jansen and Heil, 2010). In particular, they are consistent with the results of Schwarzer et al. (2012), demonstrating a link between crawling and mental rotation ability in 9-month-old infants. Our results are also congruent with the finding that manual object exploration skills are related to performance on other tasks involving spatial cognition, such as the ability to complete three-dimensional objects (Soska et al., 2010). Soska et al. demonstrated that in addition to self-sitting experience, coordinated visual-manual object explorations, such as rotation, transfers, and fingerings, significantly contributed to infant ability to complete three-dimensional objects. Similarly, Möhring and Frick (in press) provided evidence that prior manual experience with an object that was later used in a mental rotation task facilitated 6-month-olds’ mental rotation of that object. In a similar vein, but using different approaches, Needham (2000) and Perone et al. (2008) demonstrated that more skilled activity with objects altered infants’ attention to different object features.

Our findings also extend previous results on the relevance of crawling and manual object exploration for infant mental rotation ability, as they showed that the skills interact with each other. In crawling infants, the manual exploration score was not related to mental rotation ability. Independently of whether those infants manually explored the objects in the mental rotation task they looked significantly longer at the novel object. In contrast, in non-crawling infants, spontaneous exploration of the objects was associated with mental rotation ability. Infants who spontaneously explored the objects preferred to look at the novel object, similar to the infants who crawled. Thus, it seems that the experience obtained by crawling and the experience obtained by manual object exploration can mutually replace each other. This seems plausible as both experiences provide infants with the opportunity to view objects from different perspectives. Crawling around a piece of furniture, for example, or manually rotating a toy or transferring it from one hand into the other, permits the infant to detect the invariant properties of the furniture or the toy and, consequently, the infant can recognize both objects from novel perspectives.

However, infants who were not able to crawl and did not spontaneously explore the objects showed significantly different looking behavior. They looked at the familiar object for longer, instead of looking at the novel object. Moore and Johnson (2011) had also observed this type of looking behavior in a similar mental rotation task setting when they tested very young infants (3-months-old). They found that female 3-month-olds looked at the familiar and novel objects for similar durations, whereas male 3-month-olds looked significantly longer at the familiar, rather than the novel, object. The authors interpreted the infants’ familiarity preference as reflecting a cognitive or perceptual operation that was especially complex for the infants.

In future research it should be tested whether Moore and Johnson’s interpretation of infant familiarity preference can be transferred to the interpretation of familiarity preferences of the non-crawling and non-exploring infants in our study, that is, whether for those infants, the mental rotation task is a very complex task because they lack the experiences about objects obtained by crawling or manual object exploration. According to Hunter and Ames (1988) familiarity preferences following habituation will be more likely when infants have failed to complete their processing because of its complexity despite having reached the habituation criterion. In such cases, infants are thought to remain attentive to the previously seen stimulus because they are trying to obtain additional information from the stimulus that was still being processed when the habituation trials ended. Thus, future studies are needed to investigate the extent to which infants’ attention during the test phase is mainly attracted by the new rotation of the habituation object so that the infants are not able to compare rotations of the familiar object to rotations of the novel object.

A limitation of the present study is that our research design did not allow for a firm conclusion regarding how infants process the test stimuli, i.e., whether they mentally rotate the test stimuli or whether they use a process, such as structural description, to enable matching in the test phase without rotation. An empirical answer to this question needs to be addressed in future work.

Additional limitations of the present study are that we tested 9-month-old infants only, i.e., that age was held constant in our research design and that the infants were classified into those with and without crawling experience and those with much, little, or no spontaneous exploration behavior. As a result, we cannot provide data about a developmental pattern of mental rotation ability, and more importantly, it is possible that a third factor contributes to the relationship between acquisition of crawling and manual exploration skills with mental rotation ability. It could be that the crawling infants and those who spontaneously explored the objects were generally more advanced than the other infants and would display performance advantages on a wide variety of tasks. However, additional analyses showed that the crawlers and non-crawlers, as well as the explorers and non-explorers, showed similar information processing measures within the habituation phase, such as total looking times and number of trials. Thus, non-exploring and non-crawling infants were not simply slower processors. The question of whether the crawlers and manual explorers are ahead in other variables remains unanswered. An appropriate study design to overcome this limitation is an experimental design in which pre-locomotor infants are randomly assigned to receive some type of self-produced locomotor experience (Uchiyama et al., 2008) and in which infants at different ages participate in the manual exploration task.

In summary, our findings indicated that all of the infants were able to master the mental rotation task, but it was the most complex task for infants who had no crawling experience or who did not explore objects spontaneously.

## Conflict of Interest Statement

The authors declare that the research was conducted in the absence of any commercial or financial relationships that could be construed as a potential conflict of interest.
